# Jejunoileal atresia and cystic fibrosis: don’t miss it

**DOI:** 10.1186/1756-0500-5-677

**Published:** 2012-12-07

**Authors:** Carolien L Siersma, Bart L Rottier, Jan BF Hulscher, Katelijne Bouman, Margriet van Stuijvenberg

**Affiliations:** 1 Department of Pediatrics, Beatrix Children’s Hospital, CA51 Hanzeplein 1, PO Box 30001, 9700 RB, Groningen, The Netherlands; 2 Department of Pediatric Surgery, Beatrix Children’s Hospital, University Medical Centre Groningen, Groningen, The Netherlands; 3 Department of Genetics, University Medical Centre Groningen, Groningen, The Netherlands

**Keywords:** Jejunal atresia, Ileal atresia, Small bowel atresia, Cystic fibrosis

## Abstract

**Background:**

While an increased prevalence of cystic fibrosis (CF) in patients with jejunal atresia and ileal atresia (JIA) has been described previously, it still may not be a practice routine to indicate a sweat test or DNA test for CFTR mutations in newborns presenting with JIA. Leading textbooks do not mention JIA as a possible presenting clinical feature of CF. We describe two cases of JIA with a delayed diagnosis of CF (4 months [post mortem] and 19 months). This led to a retrospective review of all patients with JIA in our hospital. We hypothesised that also in the past although indicated further testing for CF had not always been performed.

**Methods:**

Over an 18-year period from January 1991 until December 2008, all cases of JIA in our centre were reviewed (n=50). We compared patients who have been tested for CF (n=18) with patients who have not been tested for CF (n=32), with respect to their patient characteristics, either by logistic regression analysis or a nonparametric test (p<0.05).

**Results:**

Of all 50 patients the proportion of infants actually tested for CF was 18 (36%). A statistical significant difference between the group of patients who were tested for CF versus the group of those who were not tested was found in a higher occurrence of postoperative bilious retention after 7 days (56% versus 25%, respectively), and postoperative complications (78% versus 34%, respectively). CF was confirmed in 4 (8%).

**Conclusion:**

Testing for CF in newborns presenting with JIA does not appear to be common practice. A timely diagnosis of CF leads to presymptomatic treatment and has beneficial effects on morbidity and mortality. CF should be tested for in all children with JIA. We recommend a sweat test for term children and CFTR DNA testing as a first step for preterm infants. Medical professional awareness may be increased if future editions of leading text books in the relevant fields should include JIA as an indication to follow an appropriate CF-diagnostic algorithm.

**Trial registration:**

Statement on reporting of a clinical trial: This article is not based on a clinical trial.

## Background

Although previous studies have presented an increased prevalence of CF in children with JIA [1-4], medical professionals working in the field of neonatology and pediatric surgery may still be unaware of this. Leading textbooks in the field do not mention this association. In cases of small bowel atresia, the presence of CF might not be considered. Until recently clinical practice in our own hospital may have been illustrated by the following two cases.

The first case was a term boy with a jejunal atresia persisted to have feeding difficulties and failure to thrive until several months after the first laparotomy. Central venous lines were required for parenteral nutrition, which provoked septic episodes. At four months of age, he died of an ongoing sepsis due to Enterobacter aerogenes and multi-organ failure. In a multidisciplinary team discussion shortly after his death CF testing was suggested by a junior pediatric pulmonologist, because of the jejunal atresia in combination with failure to thrive. The boy proved to be homozygous for the delta F508 mutation and the diagnosis CF was established. The second case was a girl who had been previously admitted to our NICU with bilious vomiting from day 2. Subsequently ileal atresia was diagnosed and surgically corrected. To treat secondary stenosis another laparotomy was needed. At the age of 19 months she was referred to the pediatric pulmonologist because of respiratory symptoms (dyspnoea). Only at that time, a sweat test was performed and the abnormal results indicated CF, which was confirmed by DNA analysis of the CFTR gene, which showed homozygosity for the delta F508 mutation.

These two cases with an unacceptable delay in diagnosing CF urged us to perform a critical in-house review of our patient care; we hypothesised that also in the past indicated further testing for CF was not always performed. We therefore performed a retrospective analysis of all cases of JIA in our hospital to assess the proportion of cases of JIA in which CF was considered, and to assess the prevalence of CF in newborns with JIA in our centre.

## Methods

Based on the availability of the hospital computerised registration system over an 18-year period from January 1991 until December 2008, all cases of JIA in our centre were retrospectively reviewed. We collected clinical baseline and follow-up data of all included patients.

With respect to the patient characteristics, we compared the group of patients who have been tested for CF with the group of patients who have not been tested for CF either by univariate logistic regression analysis (for dichotomous variables) or a nonparametric test (for continuous variables). This comparison was done to find patient characteristics associated with the doctor’s decision to test for CF, if any. Because of small numbers a multivariate analysis was not performed.

The statistical significance level was set at p=0.05.

## Results

From January 1991 until December 2008, 56 cases were identified. Six cases were excluded as they were misdiagnosed as JIA: gastroschisis (n=2); jejunal stenosis (n=1); jejunal web (n=1); intussusception (n=1); and complete small intestine necrosis (n=1). Fifty cases were included: 25 cases with jejunal atresia (50%) and 25 cases with ileal atresia (50%). The diagnosis CF was considered in 18 of 50 cases (36%), which led to 4 (8%) diagnosed cases, including the two cases presented. Three were diagnosed with a sweat test. CF was then confirmed with CFTR analysis revealing homozygous delta F508 mutation (n=2) and heterozygous delta F508/711+1G>T mutation (n=1). In one case CFTR analysis showing homozygous delta F508 mutation (n=1, post-mortem diagnosis). The national Dutch CF database was checked to rule out the possibility of children in our review with CF under treatment in another CF centre.

The patient characteristics of all cases are presented in Table 1. Comparing the group of patients who have been tested (n=18) versus the group of patients who have not been tested for CF (n=32), we found a statistical significant difference only in the occurrence of postoperative aspiration of bile-stained gastric contents via a nasogastric tube persisting for 7 days or more (56% versus 25%, respectively), and postoperative complications (78% versus 34%, respectively). CF testing was done at a median age of 0.2 (interquartile range 0.1 - 0.3) years.

**Table 1:** Patient characteristics of 50 infants with JIA

	**Cases tested for CF**	**Cases not tested for CF**
	**(n=18)**	**(n=32)**
**Birth**		
Boys	9 (50%)	16 (50%)
Gestational age (weeks)	37 (34–40)	38 (35–39)
Birth weight (grams)	2518 (2180–3169)	3020 (2300–3470)
5 minutes Apgar score	10 (8–10)	10 (8–10)
Caesarean section	4 (22%)	7 (22%)
Bile stained amniotic fluid	6 (33%)	4 (13%)
Prenatal ultrasound	9 (50%)	11 (34%)
-polyhydramnion	2	6
-hyperechoic bowels	2	0
-bowel dilation	6	9
**After birth**		
Meconium after 24 hours	5 (28%)	8 (25%)
Defaecation problems later on	3 (17%)	8 (25%)
Use of enema	1 (6%)	4 (13%)
Sticky meconium	1 (6%)	0 (0%)
Abdominal distension	15 (83%)	21 (66%)
Feeding problems / vomiting	9 (50%)	19 (59%)
Vomiting or aspiration of bile-stained gastric contents (nasogastric tube)	13 (72%)	24 (75%)
Dilated bowels (X-ray)	16 (89%)	27 (84%)
**Surgery**		
Locus of atresia		
-jejunal	11 (61%)	14 (44%)
-ileal	7 (39%)	18 (56%)
Age first surgery (days)	2 (1–3)	2 (1–4)
Stomy	3 (17%)	4 (13%)
**Postoperative phase**		
Aspiration of bile-stained gastric contents (nasogastric tube) 7 days or more after surgery	10 (56%)	8 (25%)*
Defaecation problems	5 (28%)	3 (9%)
Start enteral feeding after surgery (days)	6 (5–9)	4 (2–6)
Time between start and full enteral feeding (days)	11 (7–26)	8 (5–10)
Short bowel	2 (11%)	3 (9%)
Complications	14 (78%)	11 (34%)*
-reoperation necessary	11	8
-infection	10	8
-central venous line infection	5	3
-clinical sepsis	8	6
-pneumonia	0	0
Ventilatory support after 7 days	2 (11%)	1 (3%)
Chronic Lung Disease	1 (6%)	1 (3%)
Survival	14 (78%)	31 (97%)

Values are presented in numbers (%) or medians (interquartile ranges).

* statistically significant difference between groups (p<0.05).

Of the four cases with CF, one was diagnosed in the first month after birth, the others at 4 (case 1; the diagnosis of CF was made after the patient died), 7, and 19 (case 2) months; the median age of the diagnosis of CF was 0.5 years (interquartile range 0.2 – 1.1 years).

## Discussion

The two cases clearly illustrate that even in a tertiary university hospital year after year doctors may be unaware of the association between JIA and CF. In both cases with JIA and CF the clinical course was complicated; one of the two did not survive. The review of 50 cases with JIA showed a prevalence of CF of 8%. In only 36% of all cases with JIA the possibility of CF had been considered. The Dutch national CF database includes 96% of the known patients with CF in the Netherlands (4% of CF patients do not give their consent to be registered). The CF database was checked and no other children out of our study population were found to have CF. Based on the difference we found in the occurrence of postoperative problems we may hypothesise that doctors consider CF more frequently in those cases with a complicated postoperative course. However, the severity of the clinical course can not be used reliably as predictor of CF; a routine work-up for CF should be performed in all infants with JIA. If JIA is a presenting symptom in children with milder CF-mutations, resulting in only few mild other clinical signs and symptoms, these children might not be tested. Since not everyone has been tested for CF in our series, the prevalence number of 8% might be an underestimation.

In a population-based study in the USA Roberts et al [1] found a prevalence of CF in 11% of Caucasian newborns with JIA. Stollmann et al [2] described a group of 114 cases of JIA over a 35-year period and found that atresia was associated with CF in 8% of them. Other studies show a prevalence of CF in children with JIA of about 10% [3,4]. Our report showed a similar prevalence of 8%.

Missing the diagnosis of CF has severe implications, not only for the patient, but also for his or her family. CF is inherited in an autosomal recessive manner. Therefore parents of CF patients have a recurrence risk for further offspring with CF of 25%. There were no siblings with CF in our study. Prenatal diagnosis is possible in future pregnancies. A work-up of the family is indicated in order to detect carriers and to identify other carrier couples at risk.

In our hospital before the results of this review we have not routinely performed a work-up for CF in neonates with JIA. In many current international textbooks on medical genetics, neonatology, general paediatrics, paediatric surgery and cystic fibrosis [5-10] JIA is not mentioned as an association or a possible presenting clinical feature of CF, nor is it an indication for a sweat test. The high percentage (8% in our review) of confirmed CF in children presenting with JIA may imply that a diagnosis of CF must be considered in all cases of JIA. The results of this review have led to an increased awareness and a change in the protocol in our hospital that a CF work-up should be done for every newborn with JIA as a routine. CF should be tested for in all patients with JIA. Our protocol starts with a sweat test for term children. If indeed sufficient sweat is produced, a negative result rules out CF whereas a positive result confirms the diagnosis CF. The results are available the same day and if positive lead to DNA testing for CFTR mutations. If insufficient sweat is produced DNA testing will be done instead. In preterm infants (below 37 weeks gestational age) our first step is DNA testing for CFTR mutations because it is unlikely that sufficient sweat will be produced [11,12].

Although in 2011 a routine screening of newborns for CF has actually been implemented in the Netherlands, this procedure is designed for healthy newborns. Early diagnosis leads to presymptomatic treatment, with many studies showing several beneficial effects on morbidity and mortality [13]. A high level of suspicion should be maintained for children with JIA, even if the initial screening results are negative.

## Conclusion

There still may be insufficient awareness of the increased prevalence of CF of 8-11% in newborns with JIA. Every newborn with JIA should be tested for CF. A timely diagnosis of CF leads to presymptomatic treatment and has beneficial effects on morbidity and mortality. Medical professional awareness may increase if future editions of leading text books in the relevant fields include JIA as an indication to follow an appropriate CF-diagnostic algorithm.

## Abbreviations

CF: Cystic fibrosis; JIA: Jejunoileal atresia.

## Competing interests

All authors declare that they have no competing interests.

## Authors’ contributions

CLS conceived the study, participated in the design of the study and the data collection, and helped to draft the manuscript. BLR participated in the design of the study, supervised it and helped to draft the manuscript. JBFH participated in the design of the study, and helped to draft the manuscript. KB participated in the design of the study, and helped to draft the manuscript. MVS participated in the design of the study and the data collection, performed the statistical analysis, and drafted the final manuscript. All authors read and approved the final manuscript.
